# Effects of school-based neuromuscular training on fundamental movement skills and physical fitness in children: a systematic review

**DOI:** 10.7717/peerj.13726

**Published:** 2022-07-08

**Authors:** Junlei Lin, Ruofei Zhang, Jie Shen, Aiguo Zhou

**Affiliations:** 1 School of Strength and Conditioning Training, Beijing Sport University, Beijing, China; 2 College of Competition Sports, Beijing Sport University, Beijing, China

**Keywords:** Strength, Speed, Endurance, Motor skills, Physical education

## Abstract

**Objectives:**

The primary purpose of this review was to clarify the effects of school-based integrated neuromuscular training (INT) on fundamental movement skills and physical fitness in children. The secondary purpose was to examine whether school-based INT intervention is superior to physical education (PE) intervention in enhancing motor skills and fitness.

**Methods:**

A systematic literature search was performed in four electronic databases: PubMed, Web of Science, MEDLINE (EBSCOhost), and Cochrane Central Register of Controlled Trials. The last search was performed on December 21, 2021, and was limited to the English language, human species, and peer reviewed journals. Randomized controlled trials and cluster randomized controlled trials that examine the effects of school-based INT on motor skills and/or fitness in healthy children who were aged up to 14 years old were included. Moreover, studies included in this study should compare school-based INT-induced adaptions with those generated by PE interventions. Studies that involve athletic children and additional exercise training were excluded. The Physiotherapy Evidence Database (PEDro) scale was used to assess the quality of the study.

**Results:**

Of 1,026 studies identified, seven original trials that meet the inclusion criteria were included in this review. Based on the PEDro scale, the PEDro score of seven studies was between six and eight points with a mean score of 5.29. Among the seven studies included in this study, four studies assessed physical fitness including muscular fitness (*n* = 4), speed (*n* = 3), endurance (*n* = 2), and flexibility (*n* = 2). Three studies examined the effects of INT on postural control and three studies explored its effects on motor skills. Concerning movement competence, significant and greater improvements in postural control and fundamental motor skills were observed following school-based INT interventions compared to PE intervention in two and three studies, respectively. Regarding physical fitness, neuromuscular training significantly increased muscular fitness, speed, endurance, flexibility in three, two, one, and one studies, respectively. However, only greater improvements in muscle fitness were observed in school-based INT group compared to PE group. The main limitations of this review were the lack of descriptions of training intensity and volume and the low methodological quality of the included studies.

**Conclusion:**

This review provides evidence that school-based neuromuscular training programs are superior to PE lessons in improving postural control, fundamental motor skills and muscular strength. Therefore, INT could be incorporated into traditional physical education classes in school. **Trial registration number:** CRD42022297349.

## Introduction

Childhood has been highlighted as a critical period for the development of Fundamental Movement Skills (FMS) and physical fitness, which can be improved by participation in physical activity. Insufficient levels of physical activity can affect children’s motor competence and limit neuromuscular development. FMS can be described as the sum of stability, locomotor, and object control skills (Logan et al., 2018), which have been considered to be the primary foundations of more complex and coordinated movement (Lubans et al., 2010). The mastery of FMS helps to develop children’s healthy habits, cognition, social development, and even physical fitness (Lubans et al., 2010). It is worth noting that healthy-related fitness, such as muscular strength, speed, endurance, etc., also influences the improvement of FMS as it reflects the body’s potential and proficiency in completing high-quality motor skills (Adams, Veitch & Barnett, 2018). Physical fitness has been recognized as independent factors of chronic diseases (Ortega et al., 2008). Cardiorespiratory and muscular fitness, for example, have been associated with known and unknown risk factors underlying cardiovascular diseases (Ortega et al., 2008). Therefore, it is necessary to simultaneously improve motor skills and physical fitness in children.

Integrated neuromuscular training (INT) is referred to as a training model that included movement skills activities (*e.g.*, locomotor, stability), specific tasks (*e.g.*, object control exercises), and physical fitness exercises (*e.g.*, strength, plyometric, stability, and agility) (Myer et al., 2015). In other words, unlike other types of training, the content of INT interventions can be divided into two parts, one for general movement skills and the other for sport-specific skills (Myer et al., 2011b). INT is distinguished by a combination of physical exercise and regular short rest time, but there is no clarity on which neuromuscular training program is the most effective. It was worth noting that due to the window of opportunity, the optimal beginning to start INT is at preadolescence (Myer et al., 2011b). Thus, INT has been identified as an advanced and effective method for promoting children’s movement competence, fitness, and even athletic performance (Faigenbaum et al., 2011). INT also plays a vital role in preventing sports injury (*e.g.*, ACL). A meta-analysis investigated the effects of INT on injury prevention and demonstrated that INT can effectively decrease the risk of knee injury in youth (Emery et al., 2015). Possible reasons for injury prevention and improved fitness and motor skills following INT involved a combination of efficient cognitive processing, correct fundamental movement patterns, and force production (Moody et al., 2013; Morgan et al., 2013). Moreover, INT stimulates sensory signals and neural systems that underlie dynamic joint control to improve movement skills (Risberg et al., 2001). Thus, this type of training is widely used by elite athletes (Nagelli et al., 2021), youth athletes (Granacher et al., 2018), soldiers (Ager et al., 2019), and non-athletic children (Faigenbaum et al., 2011).

The school was a place where children spent a lot of time, and the only pillar of physical development offered in schools is physical education (PE). However, school-based PE lessons are insufficient to stimulate children’s potential. Previous studies demonstrated that INT would be most beneficial for motor skills and physical performance if it is introduced in childhood as part of PE programs (Myer et al., 2011a; Myer et al., 2015), as PE classes provide a perfect environment for children to engage in physical activities and games. Therefore, attention has been put on school-based INT, which refers to the integration of INT programs within PE lessons in school. To our knowledge, the first study on school-based INT was published in 2011 (Faigenbaum et al., 2011). The authors reported that school-based INT induced significantly greater improvements in movement skills and fitness when compared to the control group who performed the PE lessons without INT intervention (Faigenbaum et al., 2011). Then, several studies confirmed that school-based INT can enhance children’s muscular strength (Faigenbaum et al., 2011; Sindic et al., 2021), speed (Duncan, Eyre & Oxford, 2018), postural control (Guzmán-Muñoz et al., 2020), endurance (Faigenbaum et al., 2011), and FMS (Duncan, Eyre & Oxford, 2018; Duncan, Hames & Eyre, 2019; Silva-Moya et al., 2022). However, there are some contradictory findings in these studies. For example, a previous study reported that eight-week INT programs (two times per week) conducted within the first 15 min of physical class cannot significantly improve postural control in children (Faigenbaum et al., 2011). In contrast, Guzmán-Muñoz et al. (2020) reported that INT programs (four weeks, two times per week) conducted within the first 20 min PE significantly increased children’s static and dynamic postural control. Inconsistencies for flexibility (Faigenbaum et al., 2011; Sindic et al., 2021) and sprint performance (Faigenbaum et al., 2011) have also been reported.

Given the pooled effects of school-based INT on motor skills and physical fitness remain unclear, it is critical to clarify this issue and determine whether INT is superior to PE program in improving children’s health-related physical and motor skills. It is unclear to teachers whether it is necessary to use INT strategies in PE lessons. Therefore, it is necessary to synthesize the existing studies. To the best of our knowledge, this is the first systematic review to determine: (1) the effects of school-based INT programs on fundamental motor skills and physical fitness in children; (2) whether school-based INT intervention is superior to PE intervention in enhancing motor skills and fitness. The findings of this study may provide PE teachers or practitioners with essential information for the design of targeted training strategies and shed light on how to further progress.

## Materials & Methods

The present systematic review follows the guidelines of the ‘Preferred Reporting Items for Systematic Reviews and Meta-Analyses’ (PRISMA) (Page et al., 2021). The review was pre-registered on PROSPER (CRD42022297349).

### Search strategy

A systematic literature search was performed in the online databases PubMed, Web of Science, MEDLINE(EBSCOhost), and Cochrane Central Register of Controlled Trials. The last search was performed on December 21, 2021. The following search terms were selected in Boolean search syntax: (“neuromuscular training” OR “integrative neuromuscular training”) AND (“child” OR “children”) AND (“postural control” OR “motor skills” OR “skill” OR “motor” OR “performance” OR “physical fitness” OR “fitness”). The search was limited to the English language, human species, and peered journals.

### Selection criteria

According to the PICOS method (Liberati et al., 2009), studies been included in this systematic review should satisfy the following criteria: (1) Population: healthy children who were aged up to 14 years old; (2) Intervention: INT programs were performed as part of school physical education classes; (3) Comparator: physical education classes were performed by control groups in school; (4) Outcome: at least FMS and/or health-related fitness (strength, power, endurance, flexibility and speed) had reported in the study; (5) Study design: randomized controlled trials (RCTs) or cluster randomized controlled trials (CRTs). Exclusively, the studies included: (1) athletic children; (2) studies that combined INT interventions with additional exercise training.

### Data collection process and quality assessment

Two authors (S and Z) independently screened relevant studies by evaluating titles, abstracts, and full-text articles to choose eligible studies based on the pre-defined inclusion and exclusion criteria. If necessary, L was contacted to resolve the disagreements concerning the inclusion of a study. Further, information was obtained from selected studies involving study characteristics (*e.g.*, authors, published year), population (*e.g.*, age, gender), intervention (*e.g.*, duration, frequency), intervention contents (*e.g.*, strength: squat, lunge), and main outcome.

The Physiotherapy Evidence Database (PEDro) scale, which is a validity and reliability tool for study quality (Yamato et al., 2017), was used to assess the methodological quality of the studies. The PEDro scale involves 11 items, and mainly focuses on four basic methodological factors including random strategy, blind method, group comparison, and statistical analysis. The total PEDro score ranges from 0 (low quality) to 10 (high quality). According to the PEDro score, the quality of the study was classified into excellent (8–10), good (5–7), fair (3–4), and poor (< 3) (Foley et al., 2003).

### Assessment of scientific evidence

The overall scientific evidence was evaluated based on the following criteria: (a) strong evidence, the results of all studies or most studies are consistent, and inconsistency may be explained (Hillier et al., 2011); (b) weak evidence, the consistency of results was reported in fewer studies, with inconsistency reflecting genuine uncertainty (Hillier et al., 2011); (c) no evidence, no relevant studies were found.

## Results

**Figure 1:**
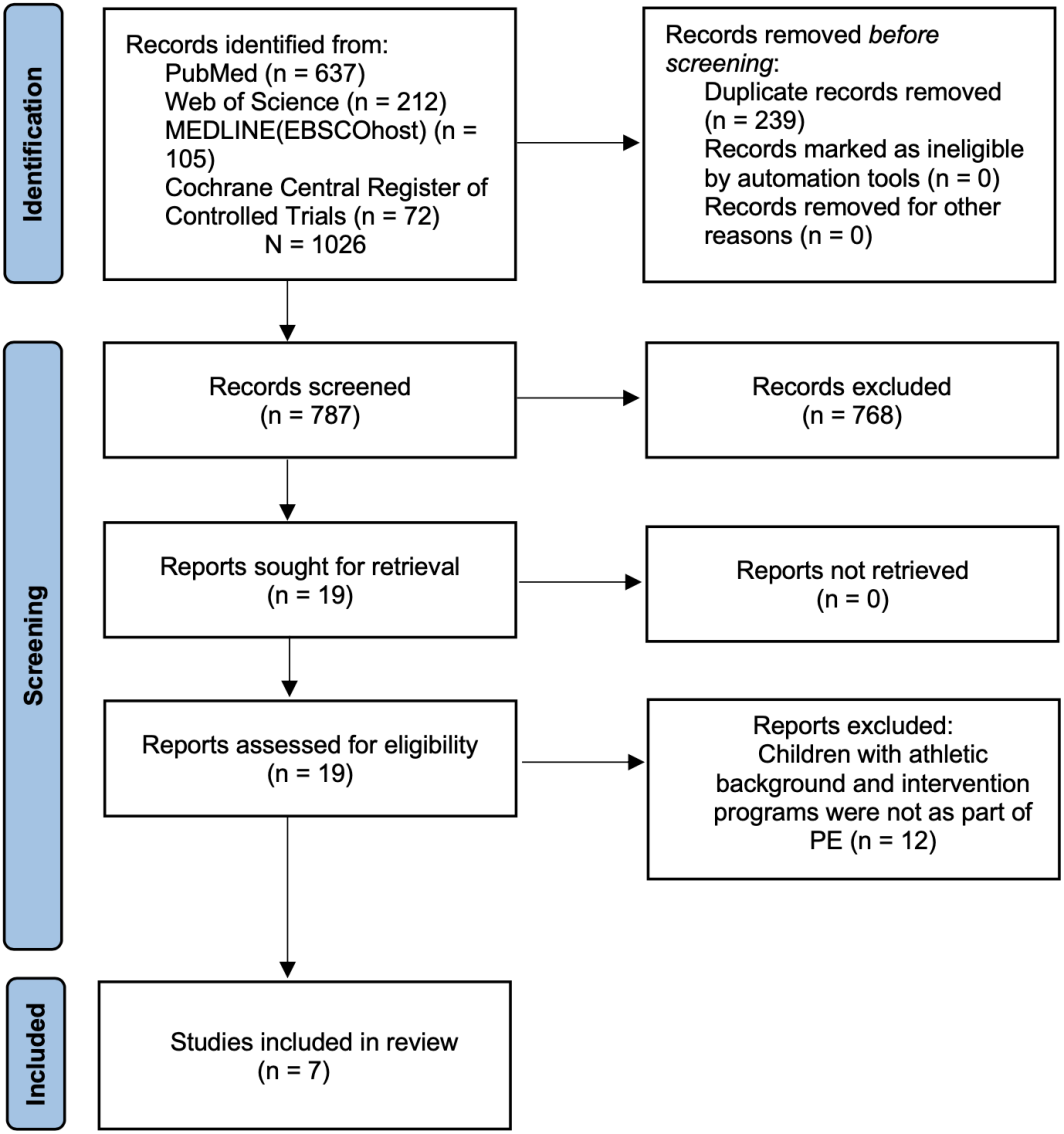
PRISMA flow chart of the study selection process.

The initial search identified a total of 1,026 potentially relevant articles (105 from MEDLINE (EBSCOhost); 637 from PubMed; 212 from Web of Science; 72 from Cochrane Central Register of Controlled Trials). Following the screening of the titles and abstracts, 768 were excluded, and then 239 duplicates were removed. The remaining 19 articles were assessed regarding the pre-defined eligibility criteria, and 12 studies were removed. Ultimately, the remaining seven original studies were included in the quantitative synthesis (Fig. 1).

### Methodological quality

The PEDro scale score of the studies is shown in Table 1. The mean PEDro score of these selected studies was 5.29, four articles were rated as good or excellent quality (5–8 points) (Sindic et al., 2021; Font-Lladó et al., 2020; Guzmán-Muñoz et al., 2020; Silva-Moya et al., 2022), and three articles were fair quality (4 points) (Duncan, Eyre & Oxford, 2018; Faigenbaum et al., 2011; Duncan, Hames & Eyre, 2019). The randomization procedure was applied in all studies. Nevertheless, all studies lacked allocation concealment and blinding therapists.

### Sample characteristics

The population characteristics of included studies are presented in Table 2. Seven studies included in this study with a total of 613 subjects (267 boys and 346 girls) ranging from 32 to 190 subjects with a mean sample size was 87.57. Six articles analyzed both boys and girls (Duncan, Eyre & Oxford, 2018; Duncan, Hames & Eyre, 2019; Faigenbaum et al., 2011; Font-Lladó et al., 2020; Guzmán-Muñoz et al., 2020; Silva-Moya et al., 2022), while one study conducted by Sindic et al. (2021) only focused on girls. The age of participants in all studies ranged from six to ten years old. One study did not report the mean age of subjects, but only children aged from eight-to ten years old could satisfy the eligibility criteria of this study (Silva-Moya et al., 2022).

### Interventions characteristics

The interventions characteristics of seven studies included in this review were provided following categories:

(1) Training period. The training period in the seven trials included in this study ranged from 4 to 12 weeks, with an average intervention period of 8.3 weeks. Two studies lasted 10 weeks (Duncan, Eyre & Oxford, 2018; Duncan, Hames & Eyre, 2019), two studies lasted 8 weeks (Faigenbaum et al., 2011; Sindic et al., 2021), and one trial lasted 12 weeks (Font-Lladó et al., 2020), 6 weeks (Silva-Moya et al., 2022), and 4 weeks (Guzmán-Muñoz et al., 2020).

(2) Training frequency. Five studies indicated that training interventions were performed twice a week (Faigenbaum et al., 2011; Font-Lladó et al., 2020; Guzmán-Muñoz et al., 2020; Silva-Moya et al., 2022; Sindic et al., 2021), whereas the other two were carried out once a week (Duncan, Eyre & Oxford, 2018; Duncan, Hames & Eyre, 2019).

(3) Duration of intervention session. Each INT session lasted between 15 min and 40 min. Specifically, the duration of each INT session was reported to last 20 min, 15 min, and 30–40 min in two (Font-Lladó et al., 2020; Silva-Moya et al., 2022), three (Faigenbaum et al., 2011; Guzmán-Muñoz et al., 2020; Sindic et al., 2021), and two studies (Duncan, Eyre & Oxford, 2018; Duncan, Hames & Eyre, 2019), respectively.

**Table 1:** Quality assessment of studies.

Authors	Sindic et al. (2021)	Faigenbaum et al. (2011)	Duncan, Eyre & Oxford (2018)	Duncan, Hames & Eyre (2019)	Font-Lladó et al. (2020)	Silva-Moya et al. (2022)	Guzmán-Muñoz et al. (2020)
Eligibility criteria	+	–	–	–	–	+	+
Random allocation	+	+	+	+	+	+	+
Concealed allocation	–	–	–	–	–	–	–
Baseline comparability	–	–	–	–	+	–	+
Blind participants	–	–	–	–	+	+	+
Blind therapists	–	–	–	–	–	–	–
Blind examiner	–	–	–	–	–	–	+
Follow-up dropout <15%	+	+	+	+	+	+	+
Intention-to- treat analysis	–	–	–	–	–	–	–
Between-group comparisons	+	+	+	+	+	+	+
Point estimates and variability	+	+	+	+	+	+	+
Score	5	4	4	4	6	6	8

**Notes.**

+ indicates a “yes” score; - indicates a “no” score.

**Table 2:** Characteristics of the included studies.

Author, year	Population	Interventions	Intervention contents	Main outcome
Font-Lladó et al. (2020)	*n* = 190 boys: 90; girls:100 age: 7.43 ± 0.32yr CG: *n* = 93, EG: *n* = 97	12 weeks, 2 times/week EG: 20 min INT warm-up + PE CG: 20 min warm-up + PE	**Strength**: squat, shoulder bridge, lunge, death lift, pull press **Plyometrics:** vertical jump, hop, lateral jump **Speed and Agility**: skip, Zig-zig run, ladder drills **Stability**: Plank, kneeling balance, bilateral or unilateral balance	motor competence: INT ↑>CG ↑ FMS: INT ↑>CG ↑
Sindic et al. (2021)	*n* = 72, girls:72 EG: *n* = 37 age: 8.17 ± 0.31yr CG: *n* = 35 age: 8.11 ± 0.31 yr	8 weeks, 2 times/week EG: 15 min INT + 30 min PE CG: 3–5 min warm-up + (10 min) stretching and strength exercises + 30 min PE	**Strength**: squat, lunge **Plyometric**: squat jump, 90° jump, hop **Object control:** catch **Stability**: plank, single leg balance, twist	push-ups: INT ↑>CG ↑ modified pull-ups: only INT ↑ flexed arm hang: only INT ↑ flexibility: both groups ↔ endurance: both groups ↔
Faigenbaum et al. (2011)	*n* = 40 boys: 16; girls: 24 EG: *n* = 21 boys:10; girls:11 age: 7.5 ± 0.3 yr CG: *n* = 19 boys:6 girls:13 age: 7.6 ± 0.3yr	8 weeks, 2 times / week EG: 15 min INT +28 min PE CG: 43 min PE	**Strength**: squat, chest press **Plyometric**: squat jump, 90° jump **Stability**: single leg balance, twist **Object control skills**: catch	push-ups: only INT ↑ curl-ups: only INT ↑ long-jump: INT ↑>CG ↑ single leg hop: INT ↑>CG ↑ 0.5-mile run: INT ↑>CG ↑ shuttle run: INT ↑ = CG ↑ stork standing: INT ↑ = CG ↑ sit and reach: INT ↑ = CG ↑
Duncan, Eyre & Oxford (2018)	*n* = 94 boys: 49, girls: 45 EG: *n* = 53 age: 6.43 ± 0.5 yr CG: *n* = 41 age: 6.23 ± 0.7 yr	10 weeks, 1 time/week EG: 30–40 min INT class CG: 30–40 min PE class	**Strength** squat, bear crawl, squat, throw **Plyometric**: hop, skip, jump **Speed and Agility**: Zig-zig run, ladder drills **Object control skills**: catch	FMS: INT ↑>CG ↑ 10 m sprint: only INT ↑ medicine ball throw: INT ↑>CG ↑ countermovement jump: only INT ↑ standing long jump: INT ↑>CG ↑
Duncan, Hames & Eyre (2019)	*n* = 140 boys: 77; girls: 63 EG 1: *n* = 50 age: 6.4 ± 0.5 yr EG 2: *n* = 48 age: 6.0 ± 0.7 yr CG: *n* = 42 age: 6.2 ± 0.5 yr	10 weeks, 1time/ week EG1 and EG 2: 30–40 min INT class CG: 30–40 min PE class	**Strength**: squat, bear crawl, squat throw, toss **Plyometric**: hop, skip, jump **Speed and Agility**: Zig-zig run, ladder drills **Object control skills**: catch	total motor competence: INT 2 ↑>INT 1 ↑>CG ↑ locomotor motor competence: both INT s ↑>CG ↑ object control motor competence: INT 2 ↑>INT 1 ↑>CG ↑ 10 m sprint: INT 2 ↑>INT 1 ↑= CG ↑ standing long jump: INT 2 ↑ >INT 1 ↑= CG ↑ seated medicine ball throw: both INTs ↑>CG ↑
Silva-Moya et al. (2022)	*n* = 45 age: 8–10 yr EG: *n* = 22 boys: 10 girls:11 CG: *n* = 23 boys: 10; girls: 13	6 weeks, 2 times/ week EG: 20 min INT + 70 min PE CG: 90 min PE	**Plyometric**: jump **Stability**: single leg standing on stable and unstable surface **Joint movement**: handheld medicine ball performing a figure **Object control:** throw ball	Balance: only INT ↑
Guzmán-Muñoz et al. (2020)	*n* = 32 EG: *n* = 16 boys:7 girls:9 age: 8.40 ± 0.72 yr CG: *n* = 16, boys:8; girls:9 age: 8.14 ± 0.77 yr	4 weeks, 2 times/ week EG: 5 min warm-up + 15 min INT + 70 min PE CG: 90 min PE	**Strength**: squat, chest press **Plyometric**: squat jump, 90° jump **Stability**: single leg balance, twist **Object control skills**: catch	Double-leg stance: only INT ↑ mSEBT: only INT ↑

**Notes.**

↑, significant within-group improvement from pretest to pos *t*-test; ↔, non-significant within-group change from pretest to pos *t*-test; >, significant and greater improvement in the former group than the latter group; =, non-significant between-group change after interventions; +, combine with; yr, year; n, number; min, minute; INT, Integrated Neuromuscular Training; EG, experimental group; CG, control group; FMS, Fundamental Movement Skills; PE, Physical Education; EG 1, Locomotor first experimental group; EG 2, Object first experimental group; mSEBT, modified Star Excursion Balance Test.

(4) Intervention content. The intervention’s primary contents involved strength (Duncan, Eyre & Oxford, 2018; Duncan, Hames & Eyre, 2019; Faigenbaum et al., 2011; Font-Lladó et al., 2020; Guzmán-Muñoz et al., 2020; Sindic et al., 2021), stability (Faigenbaum et al., 2011; Font-Lladó et al., 2020; Guzmán-Muñoz et al., 2020; Silva-Moya et al., 2022; Sindic et al., 2021), plyometric (Duncan, Eyre & Oxford, 2018; Duncan, Hames & Eyre, 2019; Faigenbaum et al., 2011; Font-Lladó et al., 2020; Guzmán-Muñoz et al., 2020; Sindic et al., 2021), object control skills (Duncan, Eyre & Oxford, 2018; Duncan, Hames & Eyre, 2019; Faigenbaum et al., 2011; Guzmán-Muñoz et al., 2020; Silva-Moya et al., 2022; Sindic et al., 2021), and speed and agility exercises (Duncan, Eyre & Oxford, 2018; Duncan, Hames & Eyre, 2019; Font-Lladó et al., 2020).

(5) Types of interventions. Five studies introduced INT into PE as warm-up session during the first 15–20 min of each traditional PE class (Faigenbaum et al., 2011; Font-Lladó et al., 2020; Guzmán-Muñoz et al., 2020; Silva-Moya et al., 2022; Sindic et al., 2021). The other two studies took the place of one of two regular PE classes per week (Duncan, Eyre & Oxford, 2018; Duncan, Hames & Eyre, 2019). Thus, although the duration of each session varied, the overall time of INT per week ranged from 30 to 40 min.

### Outcome and measures

#### Effect on postural control

Postural control was assessed in three of the seven studies selected in this review (Faigenbaum et al., 2011; Guzmán-Muñoz et al., 2020; Silva-Moya et al., 2022). The postural control was evaluated by using the stork stance test with eyes open (Faigenbaum et al., 2011), double-leg stance test with eyes open and closed (Guzmán-Muñoz et al., 2020), modified Star Excursion Balance Test (mSEBT) (Guzmán-Muñoz et al., 2020), and comprehensive balance test (including dynamic and static balance) (Silva-Moya et al., 2022).

Two studies showed significant improvements in postural control tests (Guzmán-Muñoz et al., 2020; Silva-Moya et al., 2022). Furthermore, one study found no significant changes in the control group (Guzmán-Muñoz et al., 2020), while another found greater changes in the intervention group compared with the control group (Silva-Moya et al., 2022). However, one study did not find any notable change in stork stance in the intervention and control groups (Faigenbaum et al., 2011).

#### Effect on fundamental motor skills

Among the seven studies selected in this study, three studies presented inferences about the effect of school-based INT on Fundamental motor skills (Duncan, Eyre & Oxford, 2018; Duncan, Hames & Eyre, 2019; Font-Lladó et al., 2020). The measurement tools used in these studies involved the Canadian Agility and Movement Skill Assessment (CAMSA) (Font-Lladó et al., 2020) and the Test of Gross Motor Development-2 (TGMD-2) (Duncan, Eyre & Oxford, 2018; Duncan, Hames & Eyre, 2019). The CAMSA test asked children to perform locomotor motor skills (double-foot jump, side-step, catch, overhand throw, skip, single-foot hop, and kick) while traveling 20 m (Font-Lladó et al., 2020). The TGMD-2 test included locomotor (run and jump) and object control motor (catch and throw) assessment (Duncan, Eyre & Oxford, 2018; Duncan, Hames & Eyre, 2019). Moreover, an additional bounce skill was added to assess object control skills in one study (Duncan, Eyre & Oxford, 2018).

The results of all these studies revealed that school-based INT can significantly increase movement competence (Duncan, Eyre & Oxford, 2018; Duncan, Hames & Eyre, 2019; Font-Lladó et al., 2020). Specifically, significantly greater changes were observed in the INT group compared with the control group (Duncan, Eyre & Oxford, 2018; Font-Lladó et al., 2020). Moreover, one study examined the sequencing effects of object control and locomotor skill during intervention training (Duncan, Hames & Eyre, 2019). This study revealed that the improvements in motor competence were greater for the object first group (performed object control skills before locomotor skills) than the locomotor first group (performed locomotor skills before control skills) (Duncan, Hames & Eyre, 2019). Though similar increases in locomotor motor skills were observed in both intervention groups, the object first group improved greater in object control skills than the locomotor first group (Duncan, Hames & Eyre, 2019).

#### Effect on muscular fitness

Muscular fitness was analyzed in four studies of seven studies selected in this review (Duncan, Eyre & Oxford, 2018; Duncan, Hames & Eyre, 2019; Faigenbaum et al., 2011; Sindic et al., 2021). The assessment tools used were countermovement jump (Duncan, Eyre & Oxford, 2018), standing long jump (Duncan, Eyre & Oxford, 2018; Faigenbaum et al., 2011), single-leg hop (Faigenbaum et al., 2011), curl-ups (Faigenbaum et al., 2011; Sindic et al., 2021), pull-ups (Sindic et al., 2021), push-ups (Faigenbaum et al., 2011; Sindic et al., 2021), trunk lift (Sindic et al., 2021), flexed arm hang (Sindic et al., 2021), and medicine ball throwing (Duncan, Eyre & Oxford, 2018; Duncan, Hames & Eyre, 2019).

Three studies observed significant improvements in all muscular strength tests following intervention programs (Duncan, Eyre & Oxford, 2018; Faigenbaum et al., 2011; Sindic et al., 2021). More specifically, Faigenbaum et al. (2011) reported that only the INT program significantly enhanced the curl-ups and push-up performance, and greater increases in standing long jump and single-leg hop performance were observed in the intervention group compared with the control group. Duncan, Eyre & Oxford (2018) found that INT induced greater changes in medicine ball throwing and standing long jump performance than the traditional PE program, and significant improvements in countermovement jump height were only observed in the INT group. Similarly, though performance on curl-ups, trunk lifts, and push-ups significantly increased in both groups, pull-ups and flexed arm hang performance only improved following the INT program, which also resulted in greater increases in push-ups performance compared to the control group (Sindic et al., 2021). However, one study reported that though both intervention groups significantly increased sitting medicine ball throwing performance, there was no significant difference in standing long jump between Object First intervention group and the control group (Duncan, Hames & Eyre, 2019).

#### Effect on speed

Speed performance was assessed in three studies of the seven studies selected in this study (Duncan, Eyre & Oxford, 2018; Duncan, Hames & Eyre, 2019; Faigenbaum et al., 2011). The speed test used in these studies involved a linear sprint of 10 m (Duncan, Eyre & Oxford, 2018; Duncan, Hames & Eyre, 2019) and a shuttle run of 4  × 10 yards (Faigenbaum et al., 2011). One study demonstrated that the intervention group significantly decreased 10m sprint time following school-based INT training, but not for the control group (Duncan, Eyre & Oxford, 2018).

However, Faigenbaum et al. (2011) reported notable improvement in shuttle run performance in both the intervention and control groups, but there was no significant change between both groups. Furthermore, the other study reported that a significant difference in 10 m sprint time was only observed between one of the two intervention groups and the control group, although both intervention groups improved (Duncan, Hames & Eyre, 2019).

#### Effect on endurance

Endurance performance was evaluated only in two studies of the seven studies selected in this study (Faigenbaum et al., 2011; Sindic et al., 2021). One mile run-walk test (Sindic et al., 2021) and 0.8 km run test (Faigenbaum et al., 2011) were used to assess endurance performance. One study reported that the INT program induced greater improvement in endurance performance compared to the control group, the meantime of 0.8 km run significantly reduced from 322.2 s to 298.2 s in the intervention group (Faigenbaum et al., 2011). However, there was no significant change in one-mile run-walk performance in another study (Sindic et al., 2021).

#### Effect on flexibility

Only two of seven studies included in this review study analyzed the impact of school-based INT on children’s flexibility (Faigenbaum et al., 2011; Sindic et al., 2021). The flexibility was evaluated by the sit-and-reach test (Faigenbaum et al., 2011; Sindic et al., 2021), which is generally utilized as lower back and hamstring flexibility assessment in the health population (Hui & Yuen, 2000). One study demonstrated no significant change in flexibility tests after intervention training in both groups (Sindic et al., 2021). This result is in line with the study conducted by Faigenbaum et al. (2011) who reported that significant change in flexibility was found in both intervention and control groups, but no between-group difference was evident.

## Discussion

To the best of our knowledge, this is the first systematic review that examines the effects of school-based neuromuscular training on motor skills and physical fitness in children and determines whether school-based INT intervention is superior to PE intervention. The results of seven studies indicated significant and greater improvements in children’s postural control, fundamental motor skills, and muscle strength following school-based INT intervention compared to PE lessons. However, no evidence demonstrated that school-based neuromuscular training could enhance flexibility. Cumulatively, these results provide original confirmation that school-based INT program may be superior to PE class, and significantly highlight the need to integrate INT intervention into PE lessons.

### Effect on postural control

The most important function of postural control is to establish a stable setting in which to perform specific motor tasks and to serve as a reference frame for proper exercise techniques. Two of three studies confirmed a positive effect of school-based INT on postural control (Guzmán-Muñoz et al., 2020; Sindic et al., 2021). Similarly, a previous study reported that eight-week neuromuscular training significantly increased adolescents’ mSEBT (Chaouachi et al., 2014). However, the subjects of this study were adolescents, which does not meet the criteria of this review. Proprioception and neuromuscular function are two major elements that influence postural control (Asadi, Saez de Villarreal & Arazi, 2015; Gribble & Hertel, 2003). Therefore, possible mechanisms of improved dynamic postural control may be related to positive adaptions that occurred in proprioception, muscle activation, and neuromuscular properties (Asadi, Saez de Villarreal & Arazi, 2015; Gribble & Hertel, 2003). Moreover, anticipatory adjustments that could favor muscle responses and contribute to a better motor repertoire also play an important role in the improved dynamic postural control (Aruin, Ota & Latash, 2001).

However, only one study reported no significant changes in the standing stork test after intervention (Faigenbaum et al., 2011). This could be because the authors stated that a 15-minute INT program done twice a week was insufficient to improve static stability in children (Faigenbaum et al., 2011). Moreover, of the three studies evaluating postural control, two reported greater increases in postural control in the school-based INT group compared to the control group (Guzmán-Muñoz et al., 2020; Sindic et al., 2021). Therefore, school-based INT interventions are more significant and effective in postural control than PE interventions, and the optimal dose–response of neuromuscular training for enhancing static postural control should be explored in future studies.

### Effect on fundamental motor skills

Three studies evaluated FMS (Duncan, Eyre & Oxford, 2018; Duncan, Hames & Eyre, 2019; Font-Lladó et al., 2020), which yielded significantly greater positive changes following school-based INT compared to PE programs. The results were proved by a previous study that reported that INT is an effective method to improve motor performance in soccer players (Menezes et al., 2022). These findings may also be explained by the improved neuromuscular control (Myer et al., 2011b). Repeated exposure to basic motor patterns stimulates joint mechanoreceptors and proprioceptors that induce central and peripheral neuronal adaptations that improve motor control (Asadi, Saez de Villarreal & Arazi, 2015). Thus, INT improves children’s simple movements to a large extent. This hypothesis is supported by the study undertaken by Sindic et al. (2021) who found that a six-week school-based INT resulted in significant improvements in active joint position sense.

Furthermore, one study demonstrated that greater changes in motor skills competence were observed in object first group (Duncan, Hames & Eyre, 2019). This may be explained by that the object first intervention program provided more total time and focus to develop these skills (Duncan, Hames & Eyre, 2019). Given that the school-based group improved their motor skills greater than the control group in two of the three studies assessing motor skills (Duncan, Eyre & Oxford, 2018; Font-Lladó et al., 2020). As a result, school-based INT interventions are more significant and beneficial in FMS compared with PE lessons, and it is necessary to consider the optimal sequencing of neuromuscular training programs.

### Effect on muscular fitness

Muscular fitness can be divided into muscle strength and muscle power. Two studies reported on muscle strength including upper limb and core strength, which yield positive adaptions (Faigenbaum et al., 2011; Sindic et al., 2021). Muscle strength is influenced by both neural and morphological factors (Bohannon, 1983). The development of neural variables is the most important element in boosting children’s strength performance (Ramsay et al., 1990). Therefore, improved muscle strength following intervention programs may be related to activation, coordination, recruitment, and firing of motor units (Ramsay et al., 1990). Notably, postural control, which was found to improve following school-based INT (Guzmán-Muñoz et al., 2020; Sindic et al., 2021), also had an impact on force generation (Hamed et al., 2018). For example, Saeterbakken & Fimland (2013) compared muscle strength and activity on stable and unstable surfaces. The authors found that lower 6 RM loads and triceps and pectoralis EMG activity were observed on unstable surfaces, which is difficult for subjects to maintain postural control.

Two of three studies confirmed that school-based INT programs significantly increase muscle power tests (Duncan, Eyre & Oxford, 2018; Faigenbaum et al., 2011). Enhanced fundamental motor abilities and neuromuscular control could potentially account for these changes. It is worth noting that improved maximum strength also has positive effects on power output (Andersen & Aagaard, 2006). Maximal strength shared 80% of the variance in power (Andersen & Aagaard, 2006), and improved maximal strength following intervention has a positive effect on the rate of force development characteristics (Aagaard et al., 2002).

However, one study indicated that only one of two intervention groups had a significant change in standing long jump performance compared with the control group (Duncan, Hames & Eyre, 2019). This could be explained as the authors stating that the locomotor first intervention program enabled more effort and energy put into strength and power training, resulting in larger positive muscle power adaptions (Duncan, Hames & Eyre, 2019). Unfortunately, only one study explored the sequencing effect of the INT program on muscular fitness.

Moreover, three of the four studies evaluating muscle fitness found that school-based INT interventions resulted in significant and greater improvements in muscle fitness than the PE interventions (Duncan, Eyre & Oxford, 2018; Faigenbaum et al., 2011; Sindic et al., 2021). Thus, strong evidence suggested that the INT program is more significant and effective in improving muscle fitness than PE programs. Future studies should consider the sequencing effects of neuromuscular training on power.

### Effect on speed

One study examined shuttle run performance and reported that there was no significant between-group difference in time of 4 × 10 yards following intervention programs (Faigenbaum et al., 2011). The explanation for this result may be the impact of repeated accelerations, decelerations, and changes of direction are larger in shuttle run performance.

Two studies examined linear sprint performance (Duncan, Eyre & Oxford, 2018; Duncan, Hames & Eyre, 2019), and only one study found positive adaptions following intervention (Duncan, Eyre & Oxford, 2018). This result was aligned with the results of the study by Hopper et al. (2017), in their study, the 20 m sprint time of netball players significantly improved after six-week neuromuscular training. Though the intervention type and participants of this study were not met the included criteria in this systematic review. This adaption may be due to the increases in muscular strength. Massive studies proved that there is a significant relationship between maximal strength and sprint performance (Brady et al., 2019; McBride et al., 2009), and improvements in strength after resistance training can transfer positively to acceleration performance (Seitz et al., 2014; Styles, Matthews & Comfort, 2016). In addition, improved postural control and FMS also influence force generation, which also plays an important role in sprint performance.

However, in another study, a significant difference in 10 m time was only observed between one of two intervention groups (object first group) and the control group (Duncan, Hames & Eyre, 2019). It is difficult to pick up this difference due to the lack of sufficient evidence. As a result, it is difficult to determine whether INT has a positive impact on speed ability. More studies should explore the effect of the school-based neuromuscular training program on speed and agility performance.

### Effect on endurance

Two studies included in this review evaluated endurance performance (Faigenbaum et al., 2011; Sindic et al., 2021). According to one study, school-based INT enhanced the meantime of 0.8 km run (Faigenbaum et al., 2011). This improvement may be associated with a tendency to be “metabolic nonspecialists” in children, which means that improved muscle strength and power can improve aerobic endurance (Faigenbaum et al., 2009; Safrit, 1995). It is worth mentioning that this supposition has been backed up by a previous study (Faigenbaum et al., 2009). The authors reported that nine weeks of plyometric training significantly reduced a half mile run time in 8–10 yrs children (Faigenbaum et al., 2009).

Nevertheless, another study found no significant changes in one-mile run-walk performance (Sindic et al., 2021). In contrast, the specificity of the school-based INT interventions, which included dominant strength exercises, could explain the differences in results. Therefore, it was unable to form a definite conclusion on whether the school-based INT programs are beneficial for endurance performance, research on endurance in school-based INT training must be addressed and updated.

### Effect on flexibility

The sit and reach test is a typical way to assess the flexibility in the physical fitness test. Both studies confirmed that no significant difference in sit and reach was observed between the intervention group and control group. Again, the specificity of the intervention programs may be to account for these findings, and more exercises need to be applied in school-based INT interventions (Sindic et al., 2021).

As mentioned above, this study found that the school-based INT significantly increased postural control, fundamental motor skills, and muscular strength in children, with magnitudes greater than PE intervention. A previous systematic review found that despite conflicting findings, most studies indicated that INT significantly improved postural control, coordination, and muscular strength in young athletes (Sañudo et al., 2019). Moreover, the authors stated that improved FMS may explain increases in coordination (Sañudo et al., 2019). A meta-analysis also reported that INT was effective for improving muscular fitness in children and youth with Down’s syndrome (Sugimoto et al., 2016). However, we were unsure whether INT can be beneficial for increasing speed and endurance performance due to contradictory results. Again, a systematic review conducted by Sañudo et al. (2019) demonstrated that no significant adaptations between-groups in speed were observed in most studies. Noteworthy, both previous systematic reviews focused on youth athletes or a combination of specific children and youth, with sports training, everyday activity, and other interventions employed in the control group. Although these characteristics differ from this review study, these findings supported this study’s findings. Furthermore, we found that school-based INT did not affect flexibility.

The present study has many limitations that should be addressed. Firstly, the major limitation of this systematic review was the lack of a description of the training volume and intensity. The INT programs are composed of numerous sections (resistance strength, stability, plyometrics, speed/agility, etc.), which were exposed to various training volume and intensity. Furthermore, the intervention load varies depending on the different phases. As a result, describing the exercise volume and intensity is challenging. Secondly, the methodological quality of the included studies is poor, as only three out of seven studies achieved the six points on the PEDro scale, which showed good quality research.

Despite these limitations, the strengths of this review include: (1) this is the first study to systematically synthesize available evidence of school-based INT effects on motor skills and physical fitness; (2) four major databases were searched to provide a comprehensive range of studies; (3) comprehensive search strategy follows the Preferred Reporting Items for Systematic Reviews and Meta-Analyses (PRISMA) guidelines.

## Conclusions

The main results of this systematic review were that school-based neuromuscular training programs induced significantly greater improvements in children’s postural control, fundamental motor skills, and muscular strength compared to PE interventions. However, no significant increases in flexibility performance were observed following school-based INT. Furthermore, data for endurance and speed performance were insufficient to draw firm conclusions. In conclusion, school-based INT is superior to PE lessons in improving postural control, fundamental motor skills, and muscular strength. If the purpose of the PE classes is to develop motor skills and muscle fitness, this study recommends that INT can be incorporated into PE classes. Furthermore, to obtain optimal adaptations, training components such as strength, stability, plyometrics, and object control skills training must be included based on the growth and developmental characteristics of children. Future studies are recommended to explore the effects of neuromuscular training in school-aged youth and consider the optimal exercise volume and intensity for teenage.

##  Supplemental Information

10.7717/peerj.13726/supp-1 Supplemental Information 1 Raw data

10.7717/peerj.13726/supp-2 Supplemental Information 2 PRISMA checklist

10.7717/peerj.13726/supp-3 Supplemental Information 3 The rationale for conducting the systematic review

## Additional Information and Declarations

### Data Availability

The following information was supplied regarding data availability: The instructions for collecting raw data are available in the Supplemental File.
