# Supplementary material for: Effects of school-based neuromuscular training on fundamental movement skills and physical fitness in children: a systematic review
Source: PeerJ. 2022 Jul 8;10:e13726. doi: 10.7717/peerj.13726 (PMC9272814; doi:10.7717/peerj.13726)

**Supplementary Information**

**Article title:**Effect of School-based Neuromuscular Training on Fundamental Movement Skills and Physical Fitness in Children: A Systematic Review

**Authors:**

Junlei Lin^1^, Ruofei Zhang^2^, Jie Shen^1^, Aiguo Zhou^1^

**Affiliation:**

1 School of Strength and Conditioning Training, Beijing Sport University, Beijing, China

2 School of Sport competition, Beijing Sport University, Beijing, China

**Corresponding author:**

Aiguo Zhou^1^

No. 48 Xinxi Street, Beijing, China

Email address: [296485225@qq.com](mailto:296485225@qq.com)

ORCID: https://orcid.org/0000-0003-0926-139X

**Contents:**1. Full electronic search strategy for PubMed

2. Full electronic search strategy for web of science

3. Full electronic search strategy for Medline

4. Full electronic search strategy for Cochrane ***1. Full electronic search strategy for Pubmed:***


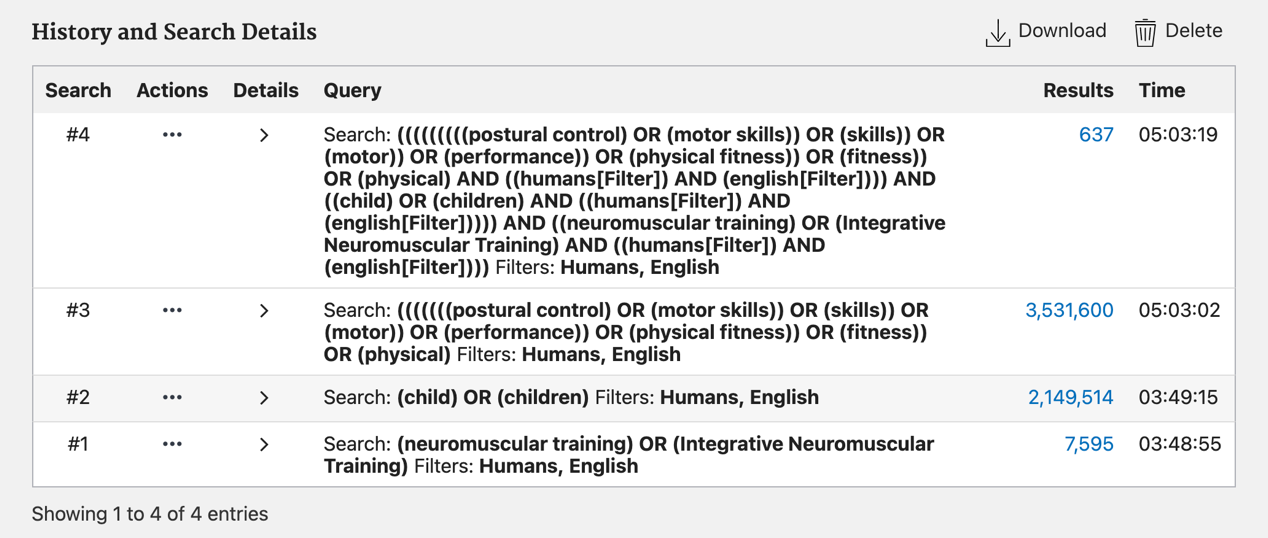


1. ***Full electronic search strategy for WoS:***


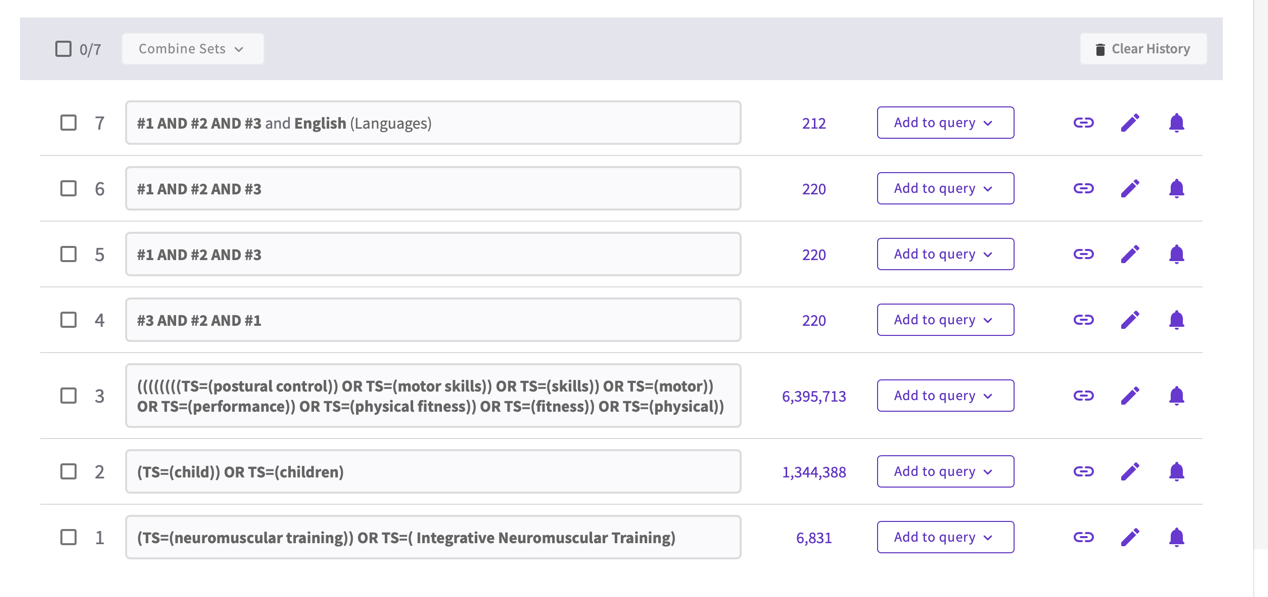


1. ***Full electronic search strategy for MEDLINE:***


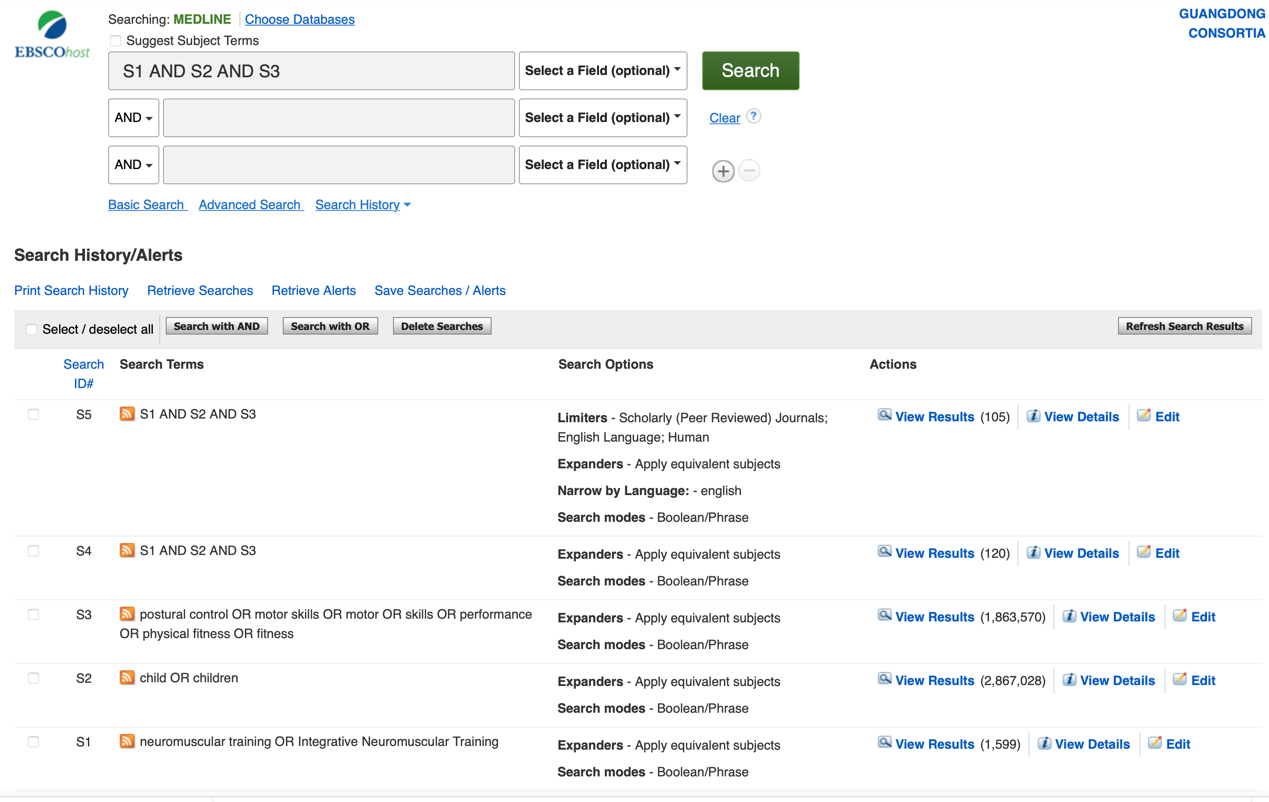


1. ***Full electronic search strategy for Cochrane:***


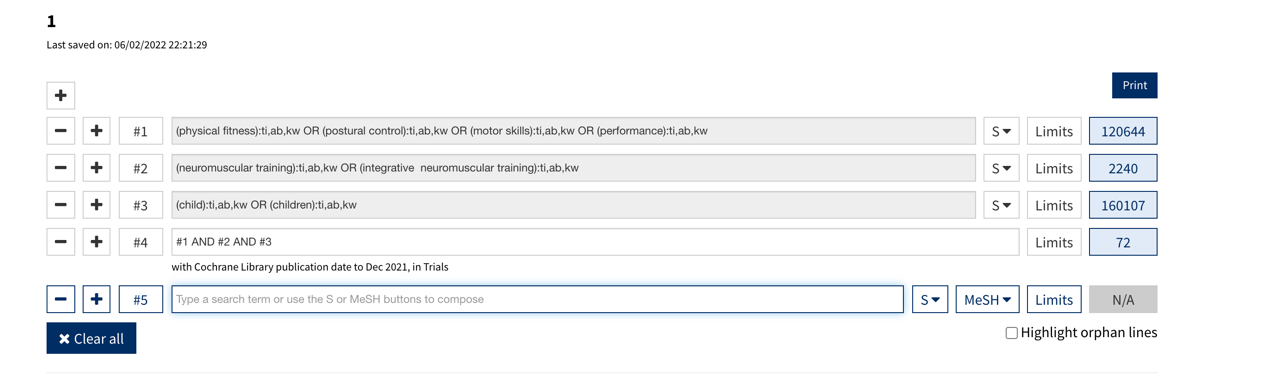

Supplement: Supplemental Information 1 [file peerj-10-13726-s001.docx]
