# Supplementary material for: Effects of school-based neuromuscular training on fundamental movement skills and physical fitness in children: a systematic review
Source: PeerJ. 2022 Jul 8;10:e13726. doi: 10.7717/peerj.13726 (PMC9272814; doi:10.7717/peerj.13726)
Supplement: Supplemental Information 3 [file peerj-10-13726-s003.docx]

The rationale for conducting the systematic review / meta-analysis:

Childhood has been highlighted as a critical development period of Fundamental Movement Skills (FMS) and physical fitness, which can be improved by participation in physical activity. Insufficient levels of physical activity can affect children’s motor competence and limit neuromuscular development. Physical fitness has been recognized as independent factors of chronic disease. Therefore, it is necessary to improve motor skills and physical fitness in children. School was a place where children spent a lot of time, and the only pillar of physical development offered in schools is Physical Education (PE). However, the traditional PE is insufficient to stimulate children’s potential. School-based neuromuscular training is frequently used to enhance children’s movement competence and physical fitness. However, there are conflicting results in previous studies regarding the effects on health-related fitness and a lack of systematic reviews on the subject. Therefore, this study aimed to clarify the effectiveness of school-based neuromuscular training on fundamental movement skills and physical fitness in children.

The contribution that it makes to knowledge in light of previously published related reports, including other meta-analyses and systematic reviews

To our knowledge, this is the first systematic review to investigate whether school-based neuromuscular training is more efficient than conventional PE for children's health-related physical fitness and motor performance. We find that school-based neuromuscular training programs induced significantly greater increases in postural control, fundamental motor skills, and muscular strength than traditional physical education class. Therefore, we suggest that school-based INT is a feasible and high effective method to increase movement competence and physical fitness.
